# Supplementary material for: Cerebrospinal fluid dynamics correlate with neurogenic claudication in lumbar spinal stenosis
Source: PLoS One. 2021 May 12;16(5):e0250742. doi: 10.1371/journal.pone.0250742 (PMC8115821; doi:10.1371/journal.pone.0250742)
Supplement: S1 Fig — (DOCX) [file pone.0250742.s002.docx]

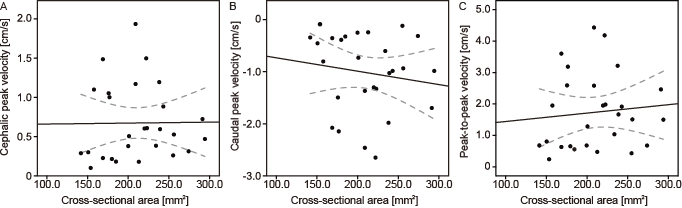


**S1 Fig.** Scatter plots of the cross-sectional area versus CSF dynamics variables. Comparison of the min AP with cephalic peak velocity (A), caudal peak velocity (B), and peak-to-peak velocity (C).
