# Supplementary material for: Cerebrospinal fluid dynamics correlate with neurogenic claudication in lumbar spinal stenosis
Source: PLoS One. 2021 May 12;16(5):e0250742. doi: 10.1371/journal.pone.0250742 (PMC8115821; doi:10.1371/journal.pone.0250742)
Supplement: S1 File — (DOCX) [file pone.0250742.s003.docx]

A univariate linear regression analysis in the elderly subjects was conducted to determine the correlations between LSS symptom severity and demographic, structural, and CSF dynamics parameters. Except for sex, all variables were significantly correlated with symptom severity as determined by claudication distance (Table). The claudication distance increased significantly with increasing age, BMI, and caudal peak velocity (age: p < 0.001, BMI: p = 0.08, and caudal peak velocity: p = 0.002). A significantly decreased claudication distance was observed in subjects with a greater minimum AP diameter, cephalic peak velocity, and peak-to-peak velocity (minimum AP diameter: p = 0.08, cephalic peak velocity: p = 0.002, and peak-to-peak velocity: p = 0.001).

**Table.** Univariate linear regression analysis to explain the claudication distance score in the elderly subjects.

|  | B (95% CI) | Adjusted R^2^ | p-value |
| --- | --- | --- | --- |
| Sex (male = 0, female = 1) | -0.277 (-1.462–0.908) | -0.036 | 0.63 |
| Age | **-0.088 (-0.15–-0.025)** | **0.251** | **<0.001** |
| Body mass index | **-0.147 (-0.314–0.019)** | **0.098** | **0.08** |
| Minimum AP diameter | **0.293 (0.08–0.507)** | **0.246** | **0.08** |
| Cephalic peak velocity | **2.129 (0.856–3.402)** | **0.335** | **0.002** |
| Caudal peak velocity | **-1.234 (-1.949–-0.519)** | **0.350** | **0.002** |
| Peak-to-peak velocity | **0.810 (0.348–1.271)** | **0.359** | **0.001** |

Bolded: statistically significant.

Minimum AP diameter: minimum anterior-posterior diameter.
