# Supplementary material for: Cerebrospinal fluid dynamics correlate with neurogenic claudication in lumbar spinal stenosis
Source: PLoS One. 2021 May 12;16(5):e0250742. doi: 10.1371/journal.pone.0250742 (PMC8115821; doi:10.1371/journal.pone.0250742)
Supplement: S2 File — (DOCX) [file pone.0250742.s004.docx]

A logistic regression analysis was conducted to determine whether the addition of the CSF dynamics parameter improves the ability to diagnose LSS. The likelihood ratio test (LRT) is derived by calculating the difference between the -2 log likelihood values for Model 1 [age, BMI] and the other models (Model 2: age, BMI, minimum AP diameter; Model 3: age, BMI, peak-to-peak velocity; Model 4: age, BMI, minimum AP diameter, peak-to-peak velocity). The differences between the -2 log likelihood values for the models (Model 1 vs. Model 2, Model 1 vs. Model 3, and Model 1 vs. Model 4) were calculated, and the p-value was examined for the chi-square using 1 degree of freedom. The LRT indicated that adding the CSF dynamics variable to a model containing the demographic variables improved model predictive ability when compared to adding the structural variable (chi-square value of Model 2: 14.41, Model 3 and 4: 19.701). The results demonstrated that there was significant improvement in the model for predicting LSS when combined with the CSF dynamics variable.

**Table.** Logistic regression analysis and -2 log likelihood ratio test to predict LSS

|  | -2 Log Likelihood | -2 Log Likelihood Ratio Test | |
| --- | --- | --- | --- |
|  |  | LR test statistic  (chi-square value) | p-value |
| Model 1 [age, BMI] | 19.701 | Baseline model | - |
| Model 2 [age, BMI, min AP] | 5.291 | 14.410 | <0.001 |
| Model 3 [age, BMI, peak-to-peak velocity] | 0 | 19.701 | <0.001 |
| Model 4 [age, BMI, min AP, peak-to-peak velocity] | 0 | 19.701 | <0.001 |
