# Supplementary material for: Cerebrospinal fluid dynamics correlate with neurogenic claudication in lumbar spinal stenosis
Source: PLoS One. 2021 May 12;16(5):e0250742. doi: 10.1371/journal.pone.0250742 (PMC8115821; doi:10.1371/journal.pone.0250742)
Supplement: S3 File — (DOCX) [file pone.0250742.s005.docx]

The ROC curve was performed to obtain the optimal cutoff value corresponding to the best classification performance (e.g., sensitivity and specificity) of models. Figure and Table showed that the model 4, including all demographic, structural, and CSF dynamics variables, was most effective in differentiating LSS patients with 100% sensitivity, specificity, and net prediction. Similar to the trend of the adjusted R^2^ value of linear regression models in the submitted manuscript, the performance of the model was improved by adding a CSF dynamics variable.


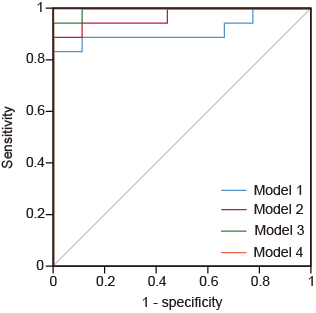


**Fig.** ROC curves for comparing healthy controls and LSS patients at the lumbar level.

**Table.** Predictive performance of CSF dynamics parameter at optimal cut-off points.

|  | Sen | Spe | PPV | NPV | LR+ | LR- | Acc | Np |
| --- | --- | --- | --- | --- | --- | --- | --- | --- |
| Model 1 [age, BMI] | 55.56 | 77.78 | 55.56 | 77.78 | 2.50 | 0.57 | 70.37 | 66.67 |
| Model 2 [age, BMI, min AP] | 77.78 | 88.89 | 77.78 | 88.89 | 7.00 | 0.25 | 85.19 | 83.33 |
| Model 3 [age, BMI, peak-to-peak velocity] | 88.89 | 94.44 | 88.89 | 94.44 | 16 | 0.12 | 92.59 | 91.67 |
| Model 4 [age, BMI, min AP, peak-to-peak velocity] | 100.00 | 100.00 | 100.00 | 100.00 | - | 0.00 | 100.00 | 100.00 |

Abbreviations: Sen=sensitivity; Spe=specificity; PPV=positive predictive value; NPV=negative predictive value; LR+=positive likelihood ratio; LR-=negative likelihood ratio; Acc=accuracy; Np=net prediction [(sensitivity + specificity)/2]. Min AP = minimum anterior-posterior diameter.
