# Supplementary material for: Cerebrospinal fluid dynamics correlate with neurogenic claudication in lumbar spinal stenosis
Source: PLoS One. 2021 May 12;16(5):e0250742. doi: 10.1371/journal.pone.0250742 (PMC8115821; doi:10.1371/journal.pone.0250742)
Supplement: Attachment — Submitted filename: Response to Reviewers.docx [file pone.0250742.s007.docx]

To the editor and reviewers of **PONE-D-21-01349R1**,

We are grateful for the reviewers’ critical comments and the editor’s decision regarding the manuscript. We recognize the importance of the advice, and we have significantly revised the manuscript to the best of our ability.

The two reviewers shared concerns regarding the ROI segmentation of the spinal canal. Given the importance of these comments, we answer the overlapping comment first and then address the questions that were more specific. Furthermore, we also employed a professional academic editing service to correct any remaining grammar or syntax problems. We consider the revised manuscript to represent a significant improvement over the previously submitted version.

**Shared concern: Region of interest (ROI) segmentation of the spinal canal**

**Comments:**

- We can see the CSF flow curves in the graph but we can’t see the Phase contrast images of the CSF flows around the spine!
- Do the ROIs include all the CSF around the spine present in the slice?
- Why a background of interest was not applied to avoid eddy current effects?
- It should also be important to know how many pixels were in the ROIs, what was the area of the ROIs ?.
- Discussion about MRI acquisition and the post processing steps is important to highlight that the measurements of the small CSF flows obtain in all the spinal canal should be interpreted with caution.

Reviewer #1

Can you show an example of an ROI placed in the dural sac?

Reviewer #2

**Answer:**

The comments above are very important. To obtain accurate CSF flows in the spinal canal, region of interest (ROI) selection must be performed with careful attention. As illustrated in the *Data analysis* section of the **Materials and Methods** section, ROI segmentation of the spinal canal was performed manually to measure CSF dynamics within the dural sac . An ROI was placed within the ventral part of the dural sac at L2 level or within the central/dorsal part at S1 level, showing an evident change in CSF flow. Then, the location of the ROI was carefully adjusted by excluding the dura mater or rootlets to ensure an accurate measurement of CSF flow velocity. The size of the rectangular ROIs was consistent among all the subjects with 9 pixels and 0.3×0.3 cm^2^. We have revised **Fig 1** by adding the phase images in the midsagittal and axial planes at the L2 and S1 levels, along with the ROI of each subject.

At the lumbosacral level, there was interruption by numerous rootlets, as shown in the typical axial image at the L2 and S1 mid-vertebral levels.

**L2**

**S1**

As above, as the spinal cord ends and divides into rootlets at the lumbosacral spine, delineating the CSF space is more challenging. In our preliminary study, CSF flow measurement erroneously alternated when the ROI included rootlets, and the CSF flow rate measured in a small area, as in this study, was parallel with the overall flow rate at the level. Thus, we chose ROIs of those small areas in the current study.

Nevertheless, we admit that the ideal ROI would be coverage of the whole intradural region while eliminating the rootlets. Following your critical comments, we have added an additional paragraph to the **Discussion** section on the limitation of CSF measurements in this study.

**Changes made in the manuscript:**

* **Fig 1** in *Data acquisition* of the **Materials and Methods** section

**Fig 1.** Observations of CSF dynamics in three typical cases: a healthy control subject (A), a healthy control subject with a narrow canal (B), and a patient with lumbar spinal stenosis (C). The 1st row depicts the T2-weighted MR images in the midsagittal plane, and the 2nd to 4th rows show the phase images in the midsagittal plane, L2 axial plane, and S1 axial plane, respectively. The 5th row shows the CSF flow velocity at the L2 (black line) and S1 (gray line) levels during one cardiac cycle. Subject A (a 56-year-old female) had no LSS symptoms and a minimum anterior-posterior diameter (min AP) of 10.38 mm at the L4/5 level. Subject B (a 61-year-old female) had no LSS symptoms but showed a narrow spinal canal with a min AP of 6.44 mm at the L4/5 level. Subject C (a 68-year-old male) had experienced neurogenic claudication for 1 year and showed multilevel stenosis at the L2/3, L3/4, and L4/5 levels with a min AP of 6.09 mm at the L4/5 level. A positive value indicates cephalic CSF flow, and a negative value denotes caudal CSF flow. The red arrowhead indicates CSF flow, and the red circle indicates the selected region of interest (ROI).

* *Data analysis* of the **Materials and Methods** section, *Line 165-166*:

“(…) The size of the rectangular ROI placed on the axial images was 0.3×0.3 cm^2^. (…)”

* 7^th^ paragraph of the **Discussion** section, *Line 340-350*:

In this study, postprocessing of the ROI segmentation has limitations in assessing the CSF flow at the lumbosacral level. The ROI was obtained manually from the small area placed within the ventral part of the dural sac. The CSF flow within the ROI could be different from the total CSF flow in the spinal canal. However, as the spinal cord ends and divides into rootlets in the lumbosacral spine, it is difficult to delineate the CSF space. Moreover, the CSF flow at the lumbosacral level is much attenuated compared with that at the cervical level; it is hard to determine the adequate threshold of the dural sac. A previous study proposed a method for semiautomated ROI segmentation at the C2-C3 level by creating a parametric image with subsequent application of a visually selected threshold [31]. Future studies may implement an automated segmentation method for CSF flow at the lumbosacral level with adequate validation.

**Specific comments from Reviewer #1**

**Comment 1:**

Authors should introduce previous pioneers works done on the CSF velocities in the spinal canal and discuss it. “Origin of subarachnoid cerebrospinal fluid pulsations: a phase-contrast MR analysis. Magn Reson Imaging. 2000 May;18(4):387-95. Henry-Feugeas MC et al”

**Answer:**

We are very grateful for the insightful advice and are confident that the comment has helped us improve the manuscript. The study conducted by Henry-Feugeas MC et al. explained that the origin of the spinal CSF pulse was mainly from intracranial pulsations. We agree with the authors and have already discussed the alteration of CSF dynamics at the brain level in the **Discussion** section; we suggest that the alteration of CSF dynamics may be influenced by pulse generation from the brain. Therefore, we have added a sentence that briefly introduced the previous study to the **Discussion** section to support our assertion.

**Changes made in the manuscript:**

* 4^th^ paragraph of the **Discussion** section, *Line 305-306*:

“(…) A previous study of CSF flow in the spinal canal reported that spinal CSF pulsations are caused mainly by intracranial pulsations [1]. (…)”

**Comment 2:**

Figure 1,2,4 present CSF flow velocity. Is it the mean flow?

- Please indicate on the graphs that positive values represent cephalic and negative caudal directions.
- If these curves represent mean velocities of the entire CSF ROIs that mean that CSF have a net flow during the cardiac cycle. In S1 mainly in the cephalic direction whereas in S2 it is mainly in the caudal direction ! IMPOSSIBLE
- If authors make the hypothesis that no net flow exist at this level and their ROIs include all the CSF around the spine, May be it should be an idea to automatically move all their CSF flow velocity curves on the Yaxis to correct the error of measurement and obtain a zero net flow. Then the results could be interpreted in a physiological way.

**Answer:**

Indeed, CSF flow velocities reported in the initially submitted manuscript were not corrected to be interpreted in a physiological way. According to your comment, CSF velocities for each subject were offset to ensure zero net flow by subtracting the mean CSF velocity during one cardiac cycle from the measured velocity. Accordingly, the CSF velocities and CSF dynamics variables, i.e., cephalic peak and caudal peak velocity, were reanalyzed. In addition, the statistical analysis of cephalic peak and caudal peak velocity was performed again. The figures and tables containing the CSF flow velocity results have been revised. Additionally, we have clarified the information on the flow direction in accordance with the value of flow velocities in **Fig 1** and its legend.

**Changes made in the manuscript:**

* *CSF velocity in patients with lumbar spinal stenosis compared with healthy controls* in the **Results** section

“(…) At the sacral level, the maximum CSF velocity tended to be lower in the patients with LSS (healthy controls vs. patients with LSS, median (interquartile range): 0.6 (0.5–0.7) vs. 0.5 (0.4–0.6) cm/s, p = 0.03), but the velocity throughout the entire cardiac cycle was not significantly different from that of the healthy controls (p = 0.62; Fig 2B). (…)”

* Figures in the **Materials and Methods,** **Results,** and **Discussion** sections

**Fig 1** has been changed as mentioned in the Shared concern.

**Table 2.** CSF flow velocity (cm/s) at L2 in the healthy controls and the patients with LSS

|  | Healthy controls | Patients with LSS | p-value* |
| --- | --- | --- | --- |
| Cephalic peak velocity | 0.8 (0.5–1.2) | 0.2 (0.2–0.3) | <0.001 |
| Caudal peak velocity | -1.3 (-2.0–-0.9) | -0.4 (-0.5–-0.3) | <0.001 |
| Peak-to-peak velocity | 1.9 (1.5–3.2) | 0.6 (0.5–0.7) | <0.001 |

* Based on the t-test.

Data represented as median (interquartile range).

**Fig 2.** Averaged CSF flow curves at the lumbosacral level over one cardiac cycle for patients with LSS and healthy controls. CSF dynamics of a patient with LSS (pink line) and a healthy control (dark blue line) at L2 (A) and S1 (B). The lines represent the mean values and the shaded areas denote the 95% confidence interval.

**Table 3.** Univariate linear regression analysis to explain the claudication distance score

|  | B (95% CI) | Adjusted R^2^ | p-value |
| --- | --- | --- | --- |
| Sex (male = 0, female = 1) | -0.385 (-1.401–0.632) | -0.015 | 0.44 |
| Age | -0.043 (-0.074–-0.012) | 0.214 | **0.009** |
| Body mass index | -0.155 (-0.290–-0.019) | 0.149 | **0.027** |
| Minimum AP diameter | 0.222 (0.044–0.400) | 0.178 | **0.016** |
| Cephalic peak velocity | 1.556 (0.678–2.434) | 0.321 | **0.001** |
| Caudal peak velocity | -1.036 (-1.586–-0.486) | 0.351 | **0.001** |
| Peak-to-peak velocity | 0.653 (0.311–0.995) | 0.358 | **0.001** |

Bolded: statistically significant.

Minimum AP diameter: minimum anterior-posterior diameter.

**Fig 3.** Scatter plots of the min AP versus CSF dynamics variables. Comparison of the min AP with the cephalic peak velocity (A), caudal peak velocity (B), and peak-to-peak velocity (C). Min AP = minimum anterior-posterior diameter.

**Fig 4.** The influence of age on the CSF flow was considered by separately comparing older (> 50 years old, light blue line) and younger (< 30 years old, blue line) healthy controls at L2 (A) and S1 (B). The lines represent the mean values and the shaded areas denote the 95% confidence interval.

**Table 6.** CSF flow velocity (cm/s) in the young and old healthy controls and patients with LSS

|  | Healthy controls | | p-value* | Patients with LSS |
| --- | --- | --- | --- | --- |
|  | Young (n = 4) | Old (n = 14) |  | (n = 9) |
| Age, years | 26 (25–28) | 58 (54–64) |  | 68 (57–75) |
| Cephalic peak velocity | 1.3 (1.0–1.8) | 0.6 (0.5–1.1) | 0.035 | 0.2 (0.2–0.3) |
| Caudal peak velocity | -2.1 (-2.4 –-1.1) | -1.2 (-1.6–-0.9) | 0.16 | -0.4 (-0.5–-0.3) |
| Peak-to-peak velocity | 3.4 (2.2–4.2) | 1.9 (1.4–2.6) | 0.046 | 0.6 (0.5–0.7) |

* Adjustment for multiple comparisons: Bonferroni.

Data represented as median (interquartile range).

* 2^nd^ paragraph of the **Discussion** section, *Line 269-271*:

“(…) Except for caudal peak velocity (r=-0.394, p=0.042), CSF dynamics variables, i.e., cephalic peak velocity and peak-to-peak velocity, had no correlation with the severity of stenosis (Fig 3). (…)”

**Comment 3:**

In the tables : simplify the numbers : example 0.7 cm/sec rather than 0.719 cm/sec unfortunately accuracy of the measurement is not so good !

**Answer:**

We regret that the initial table of CSF velocities was written poorly. The CSF velocities were refined to two significant figures, in accordance with your comments. We have revised the values in Tables 2 and 6 in the **Results** section.

**Changes made in the manuscript:**

Tables 2 and 6 have changed as mentioned in Comment #2.

**Specific comments from Reviewer #2**

**Comment 4:**

- The authors showed that there are non-negligible changes in PC-MRI measurements as a function of age; yet the LSS group is older than the control group and the control group is not normally distributed (with both young and old components).
- The linear models should be repeated with closer age matching, as univariate and multivariate linear models may be biased by bimodal distributions of variables

**Answer:**

We completely agree with you on this point. The age distribution in this study was uneven, which could induce biased results. In the submitted manuscript, to compensate for this uneven age distribution, the healthy controls were divided into young and old groups, and the CSF dynamics variables were compared between the subgroups (younger controls vs. older controls/younger controls vs. LSS patients/older controls vs. LSS patients). The CSF dynamics variables satisfied the normality condition in both the younger and older healthy controls as well as the LSS patients (the Shapiro-Wilk test was not significant, p > 0.05). Accordingly, we used the t-test for multiple comparisons between groups with Bonferroni correction (p < 0.017 (0.05/3)). As illustrated in the **Discussion** section, the CSF velocities in the older control group tended to be lower than those in the younger control group, without significance (cephalic peak velocity, p = 0.035; caudal peak velocity, p = 0.16; peak-to-peak velocity, p = 0.046). However, those variables were significantly decreased in the LSS patient group compared to both the younger and older controls. Therefore, we speculated that the reduced CSF dynamics could be regarded as a relatively discernible observation in patients with LSS with neurogenic claudication rather than a general observation in older individuals. Nevertheless, future studies involving a larger population are needed to investigate the effects of age on CSF dynamics. We have restructured the existing paragraphs in the **Discussion** section by adding a paragraph dealing with the effects of age on CSF dynamics.

In addition, to address your comment, we conducted linear regression analysis with closer age matching by excluding the younger healthy controls from all subjects (n = 23, mean age (range): 61.7 (49–76) years). Univariate and multivariate linear regression analyses were performed to determine whether the CSF hydrodynamics parameter contributed to symptom severity in the elderly subjects (S2 Table and Table 5). The predictive power was increased by the addition of the CSF dynamics parameter (model 3 [age, BMI, and peak-to-peak velocity], adjusted R^2^ = 0.623; model 4 [age, BMI, min AP, and peak-to-peak velocity], adjusted R^2^ = 0.609). Age, which showed no significance in the model for all subjects, was significantly correlated with the claudication distance score in the model for the aged subjects. Nevertheless, the predictive power of all the models showed improvements compared to that in the models analyzed in the all-subjects group (adjusted R^2^ for all subjects vs. elderly subjects, model 1: 0.262 vs. 0.359; model 2: 0.306 vs. 0.371; model 3: 0.393 vs. 0.623; model 4: 0.410 vs. 0.609). Since spinal stenosis is a degenerative disease prevalent in the elderly population, age is an important factor in neurogenic claudication, but CSF dynamics are also independently correlated with LSS symptoms. In the **Results** section, we have added the multivariate linear regression results for the elderly subjects.

**S2 Table.** Univariate linear regression analysis to explain the claudication distance score in the elderly subjects.

|  | B (95% CI) | Adjusted R^2^ | p-value |
| --- | --- | --- | --- |
| Sex (male = 0, female = 1) | -0.277 (-1.462–0.908) | -0.036 | 0.63 |
| Age | **-0.088 (-0.15–-0.025)** | **0.251** | **<0.001** |
| Body mass index | **-0.147 (-0.314–0.019)** | **0.098** | **0.08** |
| Minimum AP diameter | **0.293 (0.08–0.507)** | **0.246** | **0.08** |
| Cephalic peak velocity | **2.129 (0.856–3.402)** | **0.335** | **0.002** |
| Caudal peak velocity | **-1.234 (-1.949–-0.519)** | **0.350** | **0.002** |
| Peak-to-peak velocity | **0.810 (0.348–1.271)** | **0.359** | **0.001** |

Bolded: statistically significant.

Minimum AP diameter: minimum anterior-posterior diameter.

**Table 5.** Multivariate linear regression analysis to explain the claudication distance scores in the elderly subjects

|  | B (95% CI) | p-value | Adjusted R^2^ |
| --- | --- | --- | --- |
| *Model A (demographic variables)* | | | |
| Age | **-0.087 (-0.145–-0.028)** | **0.006** | 0.359 |
| Body mass index | **-0.144 (-0.285–-0.003)** | **0.045** |  |
| *Model B (demographic + structural variables)* | | | |
| Age | **-0.073 (-0.136–-0.011)** | **0.024** | 0.371 |
| Body mass index | -0.084 (-0.261–0.092) | 0.33 |  |
| Minimum AP diameter | 0.146 (-0.115–0.406) | 0.26 |  |
| *Model C (demographic + CSF dynamics variables)* | | | |
| Age | **-0.076 (-0.121–-0.030)** | **0.002** | 0.623 |
| Body mass index | **-0.113 (-0.222–-0.003)** | **0.045** |  |
| Peak-to-peak velocity | **0.672 (0.309–1.036)** | **0.001** |  |
| *Model D (demographic + structural + CSF dynamics variables)* | | | |
| Age | **-0.071 (-0.120–-0.021)** | **0.008** | 0.609 |
| Body mass index | -0.090 (-0.230–0.049) | 0.19 |  |
| Minimum AP diameter | 0.0580 (-0.155–0.270) | 0.58 |  |
| Peak-to-peak velocity | **0.647 (0.263–1.030)** | **0.002** |  |

Bolded: statistically significant.

Minimum AP diameter: minimum anterior-posterior diameter.

**Changes made in the manuscript:**

* *Predictive model for LSS symptom severity in elderly subjects* in the **Results** section, *Line 245-255*:

Further linear regression analysis was conducted with closer age matching by excluding the younger healthy controls from all subjects (n = 23, mean age (range): 61.7 (49–76) years; S2 File and Table 5). The predictive power was increased by the addition of the CSF dynamics parameter (model 3 [age, BMI, and peak-to-peak velocity], adjusted R^2^ = 0.623; model 4 [age, BMI, min AP, and peak-to-peak velocity], adjusted R^2^ = 0.609). Age, which showed no significance in the model for all subjects, was significantly correlated with the claudication distance score in the model for aged subjects. Nevertheless, the predictive power of all the models showed improvements compared to the models analyzed in the all-subjects group (adjusted R^2^ of the model with all subjects vs. elderly subjects, model 1: 0.262 vs. 0.359; model 2: 0.306 vs. 0.371; model 3: 0.393 vs. 0.623; model 4: 0.410 vs. 0.609).

* 3^rd^ paragraph of the **Discussion** section, *Line 280-292*:

Another possible confounder that may entail the correlation between attenuated CSF dynamics and symptom severity is age. The age distribution in this study was uneven and could lead to biased results. To compensate for the uneven distribution of age, the healthy controls were divided into younger and older groups, and the CSF dynamics variables were compared between the subgroups (younger controls vs. older controls/younger controls vs. LSS patients/older controls vs. LSS patients). The CSF velocities measured at the lumbar level in the older control group tended to be lower than those in the younger control group, without statistical significance; however, those variables were remarkably decreased in the LSS patient group compared to both the younger and older controls (Fig 4 and Table 6). The reduced CSF dynamics at the lumbar level might be regarded as a specific observation in patients with LSS with neurogenic claudication. Nevertheless, further works should be conducted with a larger population to investigate the effects of age on the attenuation of CSF dynamics.

* 6^th^ paragraph of the **Discussion** section, *Line 327-329*:

“(…) for example, in the present study, the predictive power was increased when CSF dynamics variables were added to demographic and structural variables (Tables 4 and 5). (…)”

* Supporting information

**S2 File.** Univariate linear regression analysis to explain the claudication distance score in the elderly subjects

**Comment 5:**

The venc was selected as 4 cm/s because the CSF flow was between -2 and 2 cm/s. A reference is needed for this. Also, should different velocities be thought about in each group, if CSF dynamics are significantly altered in the LSS group?

**Answer:**

We admit that the original manuscript had no reference supporting the VENC value. Very few studies have identified the specific velocities of the CSF at the lumbar level, especially in patients with LSS. Previous studies investigated CSF flows along the spinal canal (C3 to L4) in normal subjects with VENC values varying between 10 and 40 cm/s [2, 3]. In our preliminary trials, the VENC was originally set higher, and the flow was hardly apparent. After trial and error, we could adjust the VENC at 4 cm/s; we confirmed that the maximal flow rate was below 2 cm/s in the lumbar spine and thus set the VENC as twice the maximum. Additionally, we were assured that the CSF flow graphs were reproduced at VENC = 4 cm/s with satisfactory accuracy in our preliminary study. Regarding applying different VENC values to each group, a previous study demonstrated that VENC values of 10 cm/s and 50 cm/s have no significant difference in detecting CSF flow in the spinal canal [4]. Although choosing a lower VENC could increase the sensitivity for the detection of slow flow components, CSF flow could be obtained reliably even with a high VENC value. In addition, from clinical aspects, constant VENC values should be applied to all subjects to utilize PC-MRI as a diagnostic tool for LSS; a protocol for a diagnostic test cannot be distinguished based on result. We have added our pilot study, which elucidated the same VENC value used in this study, as a reference to the description in the **Materials and Methods** section.

**Changes made in the manuscript:**

* 2^nd^ paragraph of *Data acquisition* of the **Materials and Methods** section, *Line 142-144*:

“(…) velocity encoding was determined to be 4 cm/s because the velocity of the CSF vertical flow at the lumbar or sacral level was observed to range between -2 cm/s and 2 cm/s based on a pilot study [22].”

**Comment 6:**

These velocities are measured in cross sections, so since the cross-sectional areas of the dural sac in each subject are not provided, it is not clear if this could be driving the differences seen in the velocity profiles.

**Answer:**

Indeed, evaluating the cross-sectional area (CSA) is a very important issue in CSF dynamics. CSF flow could be influenced by the area of the spine. We measured the spinal canal areas at the lumbar mid-vertebral level on axial images. The CSA in the LSS patients was significantly lower than that in the healthy controls [healthy controls: 222.5 (195.0-255.3), LSS patients: 180.0 (152.5-206.5) mm^2^, median (IQR); p = 0.008]. However, when we evaluated the association between the CSA and CSF dynamics variables, none of the CSF dynamics variables were correlated with the CSA [cephalic peak velocity: r = 0.303 (p = 0.16), caudal peak velocity: r = -0.258 (p = 0.24), and peak-to-peak velocity: r = 0.312 (p = 0.11); S3 Fig]. The results suggested that structural stenosis alone could not explain the altered CSF dynamics. We have added the results of the correlation between the CSA and CSF dynamics variables to the **Discussion** section.

**S3 Fig.** Scatter plots of the cross-sectional area versus CSF dynamics variables. Comparison of the min AP with cephalic peak velocity (A), caudal peak velocity (B), and peak-to-peak velocity (C).

**Changes made in the manuscript:**

* 2^nd^ paragraph of the **Discussion** section, *Line 271-276*:

“(…) Furthermore, when we measured the cross-sectional area (CSA) at the lumbar mid-vertebral level on the axial images, the CSA in the LSS patients was significantly lower than that in the healthy controls (healthy controls vs. patients with LSS, median (interquartile range): 222.5 (195.0–255.3) vs. 180.0 (152.5–206.5) mm^2^, p = 0.008). However, none of the CSF dynamics variables were correlated with the CSA (S3 Fig).”

* Supporting information

**S3 Fig.** Scatter plots of the cross-sectional area versus CSF dynamics variables

**Comment 7:**

It can’t be said that CSF flow at the lumbar level explains the development of neurogenic claudication, but it is simply correlated with it. To explain it there has to be a definite physiological link, which, while hypothesized, has not been proven.

**Answer:**

We regret that the sentence was written rather hastily, leading to misinterpretation of our assertion. We intended to state that the CSF flow could better explain the development of neurogenic claudication than structural stenosis. The results of linear regression showed that the predictive power of neurogenic claudication was improved by the addition of CSF dynamics variables. We suggested a physiological hypothesis to explain the results in **Discussion** (line 315-321): 1) stagnant anoxia 2) impaired nutrient/oxygen supply via CSF circulation. Nevertheless, we agree that the rationale for our insistence was not sufficient to support our assertion. Following your comment, we have revised the conclusion that CSF dynamics are associated with the development of neurogenic claudication rather than explaining the symptoms.

**Changes made in the manuscript:**

* 9^th^ paragraph of the **Discussion** section, *Line 368-369*:

“To conclude, attenuation of CSF flow at lumbar spine level is associated with development of neurogenic claudication in LSS, better than severity of structural stenosis (…)”

**Comment 8:**

It is stated in line 279-280 that increased CSF pressure leads to decreased metabolic exchange. Wouldn’t an increased CSF pressure be associated with higher peak amplitudes, the opposite of what is seen in this study?

**Answer:**

As mentioned in Comment 6, CSF flow in the spine could be influenced by the various factors. Pressure gradients also determine CSF flow within the spinal canal [5] as commented. CSF pulsation is presumed to be generated from the brain. Higher pressure in the lumbosacral spine will rather reduce pressure gradient across the spine. From a hydrodynamics perspective, attenuation of CSF pulsation might have resulted from pressure differences across the spine rather than from local pressure. We speculate that LSS patients had a lower amplitude of CSF flow due to lower pressure gradients than healthy controls, despite the high CSF pressure at the stenosis level. We have revised the sentence about increased CSF pressure in LSS patients in the **Discussion** section.

**Changes made in the manuscript:**

* 5^th^ paragraph of the **Discussion** section, *Line 315-316*:

“(…) however, attenuation of CSF pulsation would be related to the pressure gradient rather than the local pressure. (…)”

**Comment 9:**

- Should an ROC analysis be used for different models?
- Table 4 – A log likelihood ratio test (LRT) would be more informative about the relative performance of each model in comparison.

**Answer:**

We greatly appreciate these insightful comments. In a previously submitted manuscript, we performed linear regression analysis to examine whether the CSF dynamics variables contributed to symptom severity, i.e., claudication distance score. An ROC analysis and a log likelihood ratio test (LRT) should be conducted to differentiate LSS patients from healthy controls instead of predicting the claudication distance score, which is a linear value.

In ROC analysis, the predicted values of the linear regression model included in the submitted manuscript were used to classify the healthy controls and LSS patients. ROC curve analysis was performed to obtain the optimal cutoff value corresponding to the best classification performance (e.g., sensitivity and specificity) of the models. S5 Fig and S5 Table show that model 4, including all demographic, structural, and CSF dynamics variables, was most effective in differentiating LSS patients with 100% sensitivity, specificity, and net prediction. Similar to the trend of the adjusted R^2^ value of linear regression models in the submitted manuscript, the performance of the model was improved by adding a CSF dynamics variable. We have provided the ROC analysis results as a supporting file (S5 File).

**S5 Fig.** ROC curves of linear regression models for comparing the healthy controls and LSS patients

**S5 Table.** Predictive performance of the CSF dynamics parameter at optimal cutoff points

|  | Sen | Spe | PPV | NPV | LR+ | LR- | Acc | Np |
| --- | --- | --- | --- | --- | --- | --- | --- | --- |
| Model 1 | 55.56 | 77.78 | 55.56 | 77.78 | 2.50 | 0.57 | 70.37 | 66.67 |
| Model 2 | 77.78 | 88.89 | 77.78 | 88.89 | 7.00 | 0.25 | 85.19 | 83.33 |
| Model 3 | 88.89 | 94.44 | 88.89 | 94.44 | 16 | 0.12 | 92.59 | 91.67 |
| Model 4 | 100.00 | 100.00 | 100.00 | 100.00 | - | 0.00 | 100.00 | 100.00 |

Abbreviations: Sen=sensitivity; Spe=specificity; PPV=positive predictive value; NPV=negative predictive value; LR+=positive likelihood ratio; LR-=negative likelihood ratio; Acc=accuracy; Np=net prediction [(sensitivity + specificity)/2]

Additionally, we conducted a logistic regression analysis to determine whether the addition of the CSF dynamics parameter improves the ability to diagnose LSS (S4 File). The likelihood ratio test (LRT) is derived by calculating the difference between the -2 log likelihood values for Model 1 [age, BMI] and the other models (Model 2: age, BMI, minimum AP diameter; Model 3: age, BMI, peak-to-peak velocity; Model 4: age, BMI, minimum AP diameter, peak-to-peak velocity). The differences between the -2 log likelihood values for the models (Model 1 vs. Model 2, Model 1 vs. Model 3, and Model 1 vs. Model 4) were calculated, and the p-value was examined for the chi-square using 1 degree of freedom. The LRT indicated that adding the CSF dynamics variable to a model containing the demographic variables improved model predictive ability when compared to adding the structural variable (chi-square value of Model 2: 14.41, Model 3 and 4: 19.701). The results demonstrated that there was significant improvement in the model for predicting LSS when combined with the CSF dynamics variable. We have provided the results of logistic regression analysis as a supporting file and added a sentence in the **Discussion** section to support the importance of CSF dynamics in LSS patients.

**S4 Table.** Logistic regression analysis and -2 log likelihood ratio test to predict LSS

|  | -2 Log Likelihood | -2 Log Likelihood Ratio Test | |
| --- | --- | --- | --- |
|  |  | LR test statistic  (chi-square value) | p-value |
| Model 1 [age, BMI] | 19.701 | Baseline model | - |
| Model 2 [age, BMI, min AP] | 5.291 | 14.410 | <0.001 |
| Model 3 [age, BMI, peak-to-peak velocity] | 0 | 19.701 | <0.001 |
| Model 4 [age, BMI, min AP, peak-to-peak velocity] | 0 | 19.701 | <0.001 |

**Changes made in the manuscript:**

* Supporting information

**S4 File.** -2 Log likelihood ratio test to determine the effect of factors on LSS

**S5 File.** ROC analysis of linear regression models to predict LSS

* 6^th^ paragraph in **Discussion** section, *Line 329-334*:

“(…) In addition, when a logistic regression analysis was conducted to determine whether the addition of CSF dynamics parameters improved the ability to diagnose LSS, adding a CSF dynamics variable was more beneficial for predicting LSS than adding structural variables (chi-square value of model 1 [age, BMI]: 14.410, model 3 [age, BMI, peak-to-peak velocity] and 4 [age, BMI, min AP, peak-to-peak velocity]: 19.701, 1 degree of freedom; p < 0.001; Table in S4 File). (…)”

**Comment 10:**

In Methods the authors stated that Bonferroni corrected p-values were accepted at p < 0.017, but in Tables 3 and 4 p-values were shown to be significant above this threshold. Were these p-values not subject to Bonferroni correction; explain why.

**Answer:**

We regret that the explanation of the Bonferroni correction was not clearly described in the *Statistical analysis* section. We applied Bonferroni correction to compare the CSF dynamics variables between the subgroups (younger controls vs. older controls/younger controls vs. LSS patients/older controls vs. LSS patients). The regression analysis results shown in Tables 3 and 4 examined the correlations between the claudication distance scores and the demographic, structural stenosis, and CSF dynamics variables. The p-values applied to linear regression analysis were used to determine the significant variables in the regression models. We have rewritten the description of the Bonferroni correction in the *Statistical analysis* in the **Materials and Methods** section.

**Changes made in the manuscript:**

* 2^nd^ paragraph of *Statistical analysis* in the **Materials and Methods** section, *Line 197-199*:

“(…) Statistical significance was set to a p-value of less than 0.05; a Bonferroni correction (p < 0.017 (0.05/3)) was applied to adjust for multiple pairwise comparisons between 3 groups.”

**Comment 11:**

- Line 43 – Define neurogenic claudication
- Line 45 – “Mechanical nerve root compression have been” should be “Mechanical nerve root compression has been”
- Line 50 – Sentence doesn’t make sense grammatically;
- Line 256-257 – This sentence doesn’t make sense
- Line 307 – Should be turbulent flow
- Fig 3 is intended to show that none of the CSF velocity profiles correlated with the severity of stenosis. There is no y labels for some plots.

**Answer:**

Thank you for your keen observations; we apologize for these errors. We admit that the initially submitted manuscript was written rather hastily, leading to several sentences and phrases that were overly verbose or grammatically troublesome. We have revised the phrasing and grammatical errors throughout the manuscript, fixed the figure errors, and employed a professional academic editing service to further address these issues. We consider the revised manuscript to be significantly improved in comparison to the previously submitted version.

**Changes made in the manuscript:**

*1^st^ paragraph of the **Introduction** section

*Line 43-45*: Neurogenic claudication is characterized by unilateral or bilateral pain during walking, standing or lumbar extension [6].

*Line 46*: Mechanical nerve root compression has been proposed for the pathophysiology of neurogenic claudication.

*Line 51*: Neurogenic claudication appears to be induced by vascular insufficiency [7-9].

*8^th^ paragraph of the **Discussion** section, *Line 359:*

Fourth, due to the technical constraints of PC-MRI, the detailed parameters of fluid dynamics (e.g., turbulent flow) were not considered.

*******3^rd^ paragraph of the **Discussion** section, *Line 288-290*:

“(…) The reduced CSF dynamics at the lumbar level might be regarded as a specific observation in patients with LSS with neurogenic claudication. (…)”

*******Fig 3 in the **Discussion** section

**Fig 3.** Scatter plots of the min AP versus CSF dynamics variables. Comparison of the min AP with the cephalic peak velocity (A), caudal peak velocity (B), and peak-to-peak velocity (C). Min AP = minimum anterior-posterior diameter.

**References**

1.	Henry–Feugeas M-C, Idy–Peretti I, Baledent O, Poncelet–Didon A, Zannoli G, Bittoun J, et al. Origin of subarachnoid cerebrospinal fluid pulsations: a phase-contrast MR analysis. Magnetic resonance imaging. 2000;18(4):387-95.

2.	Dreha-Kulaczewski S, Konopka M, Joseph AA, Kollmeier J, Merboldt K-D, Ludwig H-C, et al. Respiration and the watershed of spinal CSF flow in humans. Scientific reports. 2018;8(1):1-7.

3.	Aktas G, Kollmeier JM, Joseph AA, Merboldt K-D, Ludwig H-C, Gärtner J, et al. Spinal CSF flow in response to forced thoracic and abdominal respiration. Fluids and Barriers of the CNS. 2019;16(1):10.

4.	Knobloch V, Binter C, Kurtcuoglu V, Kozerke S. Arterial, venous, and cerebrospinal fluid flow: simultaneous assessment with Bayesian multipoint velocity-encoded MR imaging. Radiology. 2014;270(2):566-73.

5.	Yamada S, Kelly E, editors. Cerebrospinal fluid dynamics and the pathophysiology of hydrocephalus: new concepts. Seminars in Ultrasound, CT and MRI; 2016: Elsevier.

6.	Katz JN, Harris MB. Lumbar spinal stenosis. New England Journal of Medicine. 2008;358(8):818-25.

7.	Matsudaira K, Seichi A, Kunogi J, Yamazaki T, Kobayashi A, Anamizu Y, et al. The efficacy of prostaglandin E1 derivative in patients with lumbar spinal stenosis. Spine. 2009;34(2):115-20. doi: 10.1097/BRS.0b013e31818f924d.

8.	Kobayashi S, Baba H, Uchida K, Kokubo Y, Yayama T, Takeno K, et al. Blood circulation of cauda equina and nerve root. Clin Calcium. 2005;15(3):63-72. doi: CliCa0503383392

9.	Porter RW. Spinal stenosis and neurogenic claudication. Spine. 1996;21(17):2046-52. doi: 10.1097/00007632-199609010-00024.
